# Specnuezhenide Decreases Interleukin-1β-Induced Inflammation in Rat Chondrocytes and Reduces Joint Destruction in Osteoarthritic Rats

**DOI:** 10.3389/fphar.2018.00700

**Published:** 2018-06-28

**Authors:** Chiyuan Ma, Xiaopeng Zhou, Kai Xu, Linyan Wang, Yute Yang, Wei Wang, An Liu, Jisheng Ran, Shigui Yan, Haobo Wu, Lidong Wu

**Affiliations:** ^1^Department of Orthopedics Surgery, The Second Affiliated Hospital, Zhejiang University School of Medicine, Hangzhou, China; ^2^Department of Ophthalmology, The Second Affiliated Hospital, Zhejiang University School of Medicine, Hangzhou, China

**Keywords:** specnuezhenide, osteoarthritis, chondrocyte, NF-κB, wnt/β-catenin

## Abstract

As a chronic disease, osteoarthritis (OA) leads to the degradation of both cartilage and subchondral bone, its development being mediated by proinflammatory cytokines like interleukin-1β. In the present study, the anti-inflammatory effect of specnuezhenide (SPN) in OA and its underlying mechanism were studied *in vitro* and *in vivo.* The results showed that SPN decreases the expression of cartilage matrix-degrading enzymes and the activation of NF-κB and wnt/β-catenin signaling, and increases chondrocyte-specific gene expression in IL-1β-induced inflammation in chondrocytes. Furthermore, SPN treatment prevents the degeneration of both cartilage and subchondral bone in a rat model of OA. To the best of our knowledge, this study is the first to report that SPN decreases interleukin-1β-induced inflammation in rat chondrocytes by inhibiting the activation of the NF-κB and wnt/β-catenin pathways, and, thus, has therapeutic potential in the treatment of OA.

## Introduction

Osteoarthritis is one of the most common degenerative joint diseases, which involves cartilage loss, synovial inflammation and chronic pain (Benito et al., 2005; Loeuille et al., 2005). Genetic and environmental factors, including sex, obesity, and injuries, could increase the risk of developing OA (Blagojevic et al., 2010). These factors can repeatedly disrupt the joint microenvironment, leading to its degeneration. The smooth functioning of joints is ensured by cartilages at the articular surface, which rely on the tensile strength of matrix induced by chondrocytes (Chen et al., 2002). Chondrocytes take part in the synthesis and degradation of cartilage matrix, and an inflammatory microenvironment could damage this function of chondrocytes (Liu-Bryan and Terkeltaub, 2015). The inflammatory cytokines IL-1β and TNF-α are key players in OA. The stimulation of these cytokines enhances the degeneration of cartilage matrix by up-regulating matrix-degrading enzymes and down-regulating chondrocyte-specific proteins (Tetlow et al., 2001; Loeser, 2006). Dysfunction and degradation of cartilages then causes loss of smooth mobility of joints and, consequently, OA. Pathways like NF-κB and wnt/β-catenin are recognized to get involved in this progress, and activation of those pathways is related to chondrocyte-specific gene degradation and cartilage matrix-degrading enzyme expression like collagenases and MMPs in OA development (Kawaguchi, 2009; Olivotto et al., 2015; Cheleschi et al., 2017). Targeting the inflammatory progress may lead to new therapies.

Recently, plant-extracted agents have received great interest for their potential in the treatment of OA. SPN (Chemical Abstracts Service number 39011-92-2, C31H42O17, molecular weight, 686.62) is an extracted agent of the fruits of *Ligustrum lucidum*. Previous studies have already indicated the suppressive effect of SPN on certain inflammation-related signaling pathways, including MAPK/ERK and HIF-1α/VEGF (Fu et al., 2010; Wu et al., 2016). However, as SPN has only been discovered and investigated over the past few decades, its pharmaceutical value is still unclear (Fu et al., 2010). In the present study, we report, for the first time, that SPN exerts anti-inflammatory properties in a rat model of OA, and inhibits IL-1β-induced inflammation in chondrocytes by reducing NF-κB and wnt/β-catenin signaling *in vitro*.

## Results

### Effects of SPN on Rat Chondrocyte Viability and Gene Expression *in Vitro*

**FIGURE 1:**
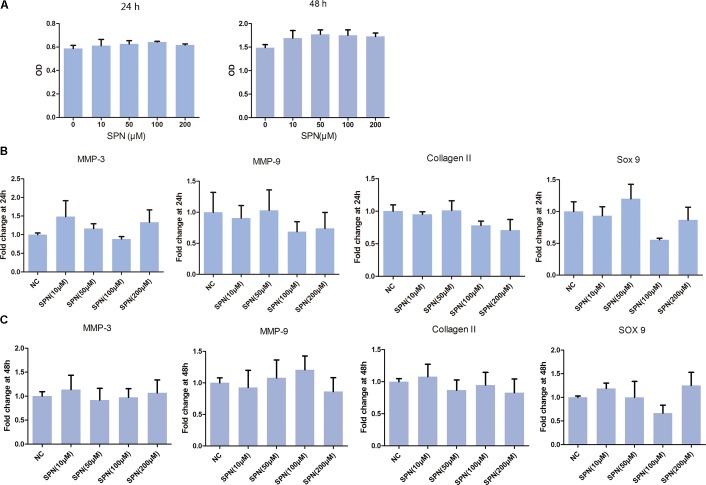
Effect of SPN on viability and gene expression of rat chondrocytes. Rat chondrocytes (5000/well) were seed in 96-well plates for 24 h and 48 h with different concentrations of SPN. **(A)** Cell viability (24 h and 48 h) was assessed by CCK-8 assay (*n* = 10 per group). **(B,C)** Effect of SPN alone (24 h and 48 h) on expression of MMP3, MMP9, collagen II, and sox9 was evaluated by real-time PCR (*n* = 5 per group).

Cytotoxicity of SPN on rat chondrocyte was evaluated by CCK-8 assay. As illustrated in **Figure 1**, SPN did not induce significant cytotoxicity in rat chondrocytes at concentrations of 0–200 μM, after 24 h and 48 h incubation. Thus, SPN was used in the following experiments at concentrations of 10, 50, 100, and 200 μM.

Subsequently, the effect of SPN on chondrocyte-specific gene expression in rat chondrocytes was observed. Chondrocytes were treated with SPN at different concentrations (10, 50, 100, 200 μM), for 24 h and 48 h, without IL-1β. As shown in **Figure 1**, SPN treatment did not influence the expression of collagen II and sox9. Moreover, the expression of cartilage matrix-degrading enzymes, such as MMP-3 and MMP-9, did not show significant changes. These results indicate that SPN treatment does not significantly impact rat chondrocyte viability or gene expression.

### Effect of SPN on IL-1β-Induced Chondrocyte-Specific Gene Degradation and Cartilage Matrix-Degrading Enzyme Expression in Rat Chondrocytes *in Vitro*

**FIGURE 2:**
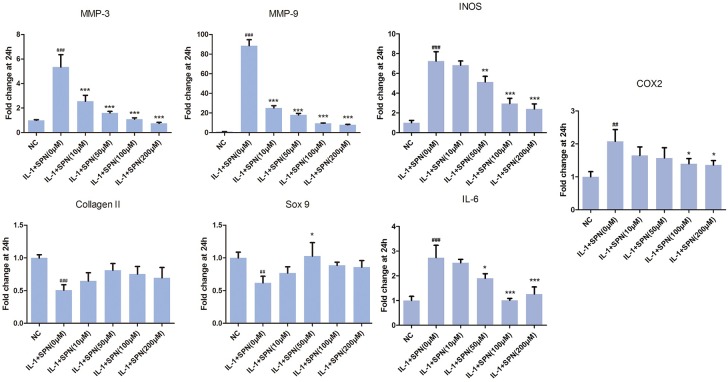
Effects of SPN on IL-1β-induced chondrocyte-specific gene degradation and mRNA levels of cartilage matrix-degrading enzymes in rat chondrocytes. Chondrocytes were treated with different concentrations of SPN for 24 h, with or without IL-1β (5 ng/ml). The mRNA levels of chondrocyte-specific genes and matrix-degrading enzymes were evaluated by Real-Time PCR (*n* = 5 per group). Significance was calculated by one-way ANOVA with *post hoc* Tukey’s multiple comparisons test. ^∗^*p* < 0.05, ^∗∗^*p* < 0.01, ^∗∗∗^*p* < 0.001 *versus* the IL-1+SPN (0 μM) group. ^##^*p* < 0.01, ^###^*p* < 0.001 *versus* the NC group.

**FIGURE 3:**
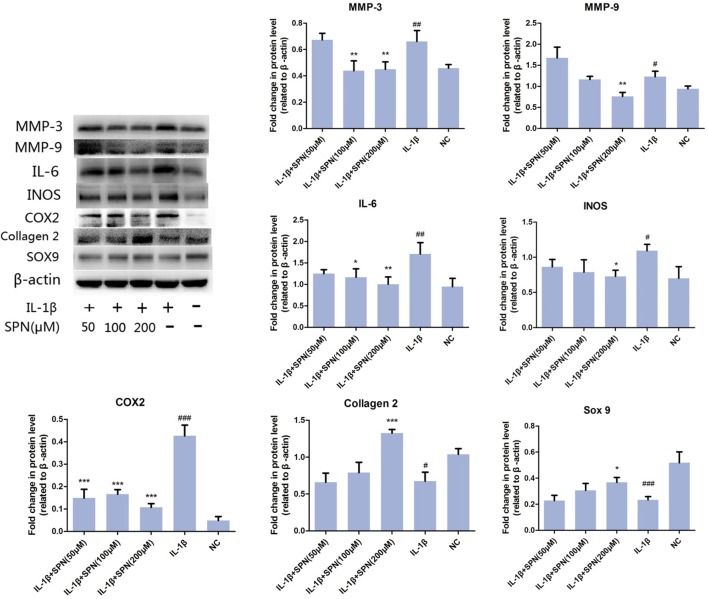
Effect of SPN on protein level of IL-1β-induced chondrocyte-specific gene degradation and cartilage matrix-degrading enzymes in rat chondrocytes. Chondrocytes were treated with different concentrations of SPN for 48 h, with or without IL-1β (5 ng/ml). The protein levels of chondrocyte-specific gene degradation and matrix-degrading enzymes were evaluated by Western blots (*n* = 5 per group). Significance was calculated by one-way ANOVA with *post hoc* Tukey’s multiple comparisons test. ^∗^*p* < 0.05, ^∗∗^*p* < 0.01, ^∗∗∗^*p* < 0.001 *versus* the IL-1+SPN (0 μM) group. ^#^*p* < 0.05, ^##^*p* < 0.01, ^###^*p* < 0.001 *versus* the NC group.

Next, we investigated the effect of SPN on IL-1β-induced chondrocyte-specific gene degradation and cartilage matrix-degrading enzyme expression in rat chondrocytes. For mRNA evaluation, chondrocytes were pretreated with SPN at different concentrations (0, 10, 50, 100, 200 μM) for 1 h, then incubated with IL-1β (5 ng/ml) for 24 h. In the present study, the mRNA expression of MMP3, MMP9, IL-6, iNOS, COX2, collagen II, and sox9 was evaluated by Real-Time PCR. As shown in **Figure 2**, the expression of MMP3, MMP9, IL-6, iNOS, and COX2 was down-regulated by SPN treatment, but the pretreatment did not significantly improve chondrocyte-specific gene degradation. For protein evaluation, chondrocytes were pretreated with SPN at different concentrations (0, 50, 100, 200 μM) for 1 h, and then incubated with IL-1β (5 ng/ml) for 48 h. Cells were harvested for Western blotting post-treatment. Protein levels of MMP3, MMP9, IL-6, iNOS, COX2, collagen II, and sox9 were evaluated by Western blots (**Figure 3**). Results showed that the expression of COX2 was down-regulated by all SPN treatments. In addition, a significant decrease in protein levels of MMP3, MMP9, IL-6, and iNOS was observed in the high-concentration groups (200 μM). Furthermore, a significant increase in protein levels of collagen II and sox9 was only observed in the 200 μM SPN treated groups. Considering iNOS and COX2 are also expressed in macrophages, and OA is an inflammation process, we investigated effect of SPN treatment on LPS-induced inflammation in RAW264.7 cells. RAW264.7 cells were pretreated with SPN at different concentrations (0, 50, 100, 200 μM) for 1 h, then incubated with or without LPS (10 ng/ml) for 24 h before analysis. As illustrated in Supplementary Figure S1, both mRNA and protein levels of iNOS and COX2 were significantly down-regulated by SPN treatment. According to the results, SPN treatment can protect the cartilage by up-regulating IL-1β-induced chondrocyte-specific genes and by decreasing the protein levels of MMPs, IL-6, iNOS, and COX2 in a dose-dependent manner *in vitro*.

### Effect of SPN on IL-1β-Induced Activation of NF-κB and Wnt/β-Catenin Signaling in Rat Chondrocytes *in Vitro*

**FIGURE 4:**
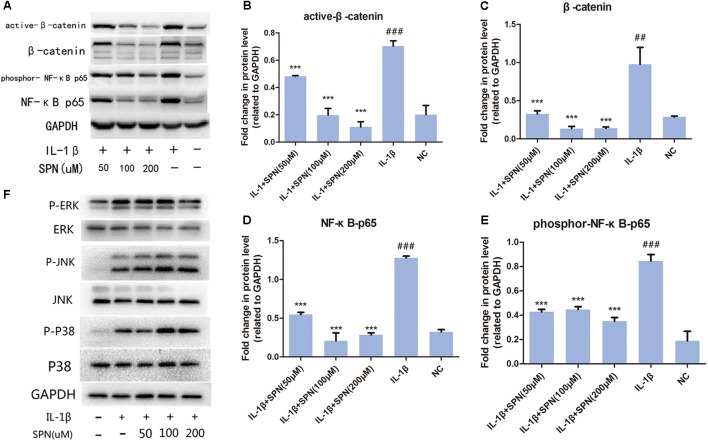
Effect of SPN on IL-1β-induced activation of NF-κB and wnt/β-catenin signaling in rat chondrocytes. **(A–E)** Chondrocytes were treated with different concentrations of SPN for 6 h, with or without IL-1β (5 ng/ml). The levels of active-β-catenin, β-catenin, NF-κB p65, and GAPDH, as well as the phosphorylation level of NF-κB p65 were assessed by Western blotting. GAPDH was used as an endogenous control (*n* = 3 per group). **(F)** Chondrocytes were pretreated with different concentrations of SPN for 2 h, and then incubated with or without IL-1β (5 ng/ml) for 30 min. The levels of ERK, JNK, and P38, as well as their phosphorylation levels were assessed by Western blotting (*n* = 3 per group). Significance was calculated by one-way ANOVA with *post hoc* Tukey’s multiple comparisons test. ^∗∗∗^*p* < 0.001 *versus* the IL-1β+SPN (0 μM) group. ^##^*p* < 0.01, ^###^*p* < 0.001 *versus* the NC group.

**FIGURE 5:**
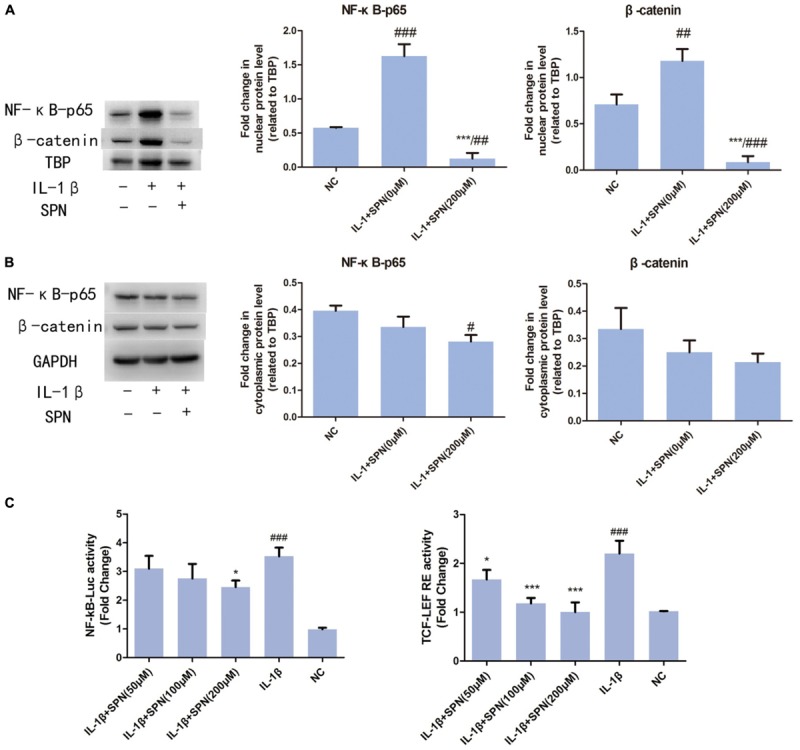
Effect of SPN on IL-1β-induced nuclear translocation of NF-κB p65 and β-catenin. Nuclear and cytoplasmic extraction reagents were used to prepare nuclear **(A)** and cytoplasmic **(B)** extracts. Then, protein levels of β-catenin, NF-κB p65, GAPDH in the cytoplasm, and β-catenin, NF-κB p65, and TBP in the nucleus were assessed by Western blotting. GAPDH was used as an endogenous control in the cytoplasm, whereas TBP worked as an endogenous control in the nucleus (*n* = 3 per group). **(C)** Chondrocytes were pretreated with SPN for 1 h, and then stimulated with IL-1β for 6 h for NF-κb-Luc activity analysis, or 12 h for TCF-LEF RE activity analysis (*n* = 5 per group). Significance was calculated by one-way ANOVA with *post hoc* Tukey’s multiple comparisons test. ^∗^*p* < 0.05, ^∗∗∗^*p* < 0.001 *versus* the IL-1β+SPN (0 μM) group. ^#^*p* < 0.05, ^##^*p* < 0.01, ^###^*p* < 0.001 *versus* the NC group.

To understand the anti-inflammatory mechanism of SPN, we investigated its effects on the IL-1β-induced activation of NF-κB and wnt/β-catenin signaling in rat chondrocytes *in vitro*, by Western blotting. The phosphorylation and protein level of NF-κB p65 in the cells were evaluated to assess the activation of NF-κB signaling, whereas the levels of active-β-catenin and β-catenin were investigated to assess the activation of wnt/β-catenin signaling. Chondrocytes were treated with different concentrations (0, 50, 100, 200 μM) of SPN for 6 h, with or without the presence of IL-1β (5 ng/ml). **Figures 4A–E** shows that the increasing levels of NF-κB-p65 and β-catenin could be inhibited by SPN treatment. Meanwhile, we also investigated the effect of SPN on IL-1β-induced activation of MAPK pathways. Chondrocytes were pretreated with SPN for 2 h, and then incubated with or without IL-1β (5 ng/ml) for 30 min. The levels of ERK, JNK, and P38, as well as their phosphorylation levels, were assessed by Western blotting (**Figure 4F**). The data indicate that SPN exerted an inhibitory effect on IL-1β-induced phosphorylation, increasing NF-κB-p65 and β-catenin in rat chondrocytes, while no inhibition of the MAPK pathway was observed. Furthermore, the effect of SPN on the transcriptional activity of NF-κb and β-catenin induced by IL-1β was examined using a luciferase reporter gene assay. Chondrocytes were pretreated with different concentrations (0, 50, 100, 200 μM) of SPN for 1 h, and then stimulated with IL-1β (5 ng/ml) for 6 h for NF-κb-Luc activity analysis, or 12 h for TCF-LEF RE activity analysis. The luciferase reporter gene assay showed that the IL-1β-induced transcriptional activity of the NF-κb and wnt/β-catenin pathways was greatly decreased by treatment with SPN (**Figure 5C**).

**FIGURE 6:**
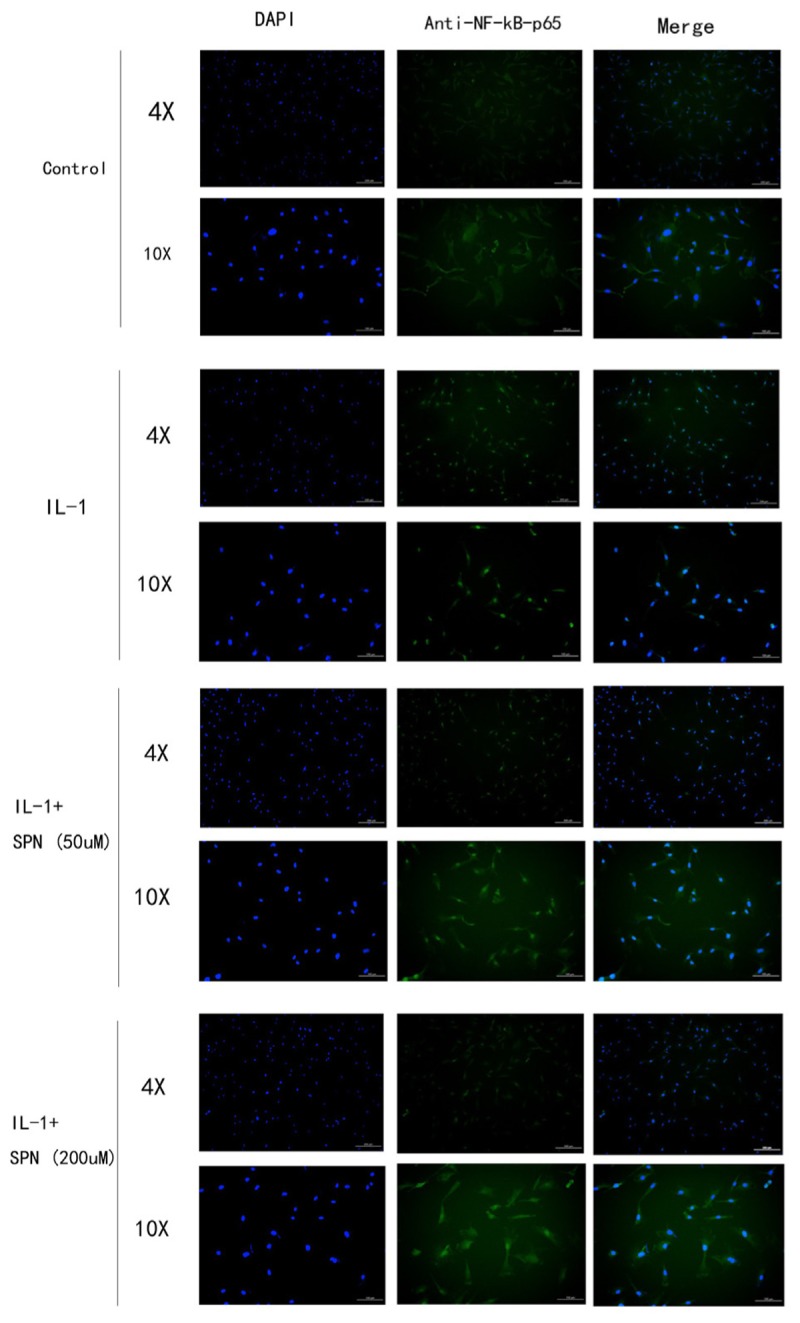
The location and concentration of NF-kB-p65 (green) was visualized using immunofluorescence microscopic analysis. Chondrocytes were treated with different concentrations of SPN for 30 min, with or without IL-1β (5 ng/ml) (*n* = 3 per group).

**FIGURE 7:**
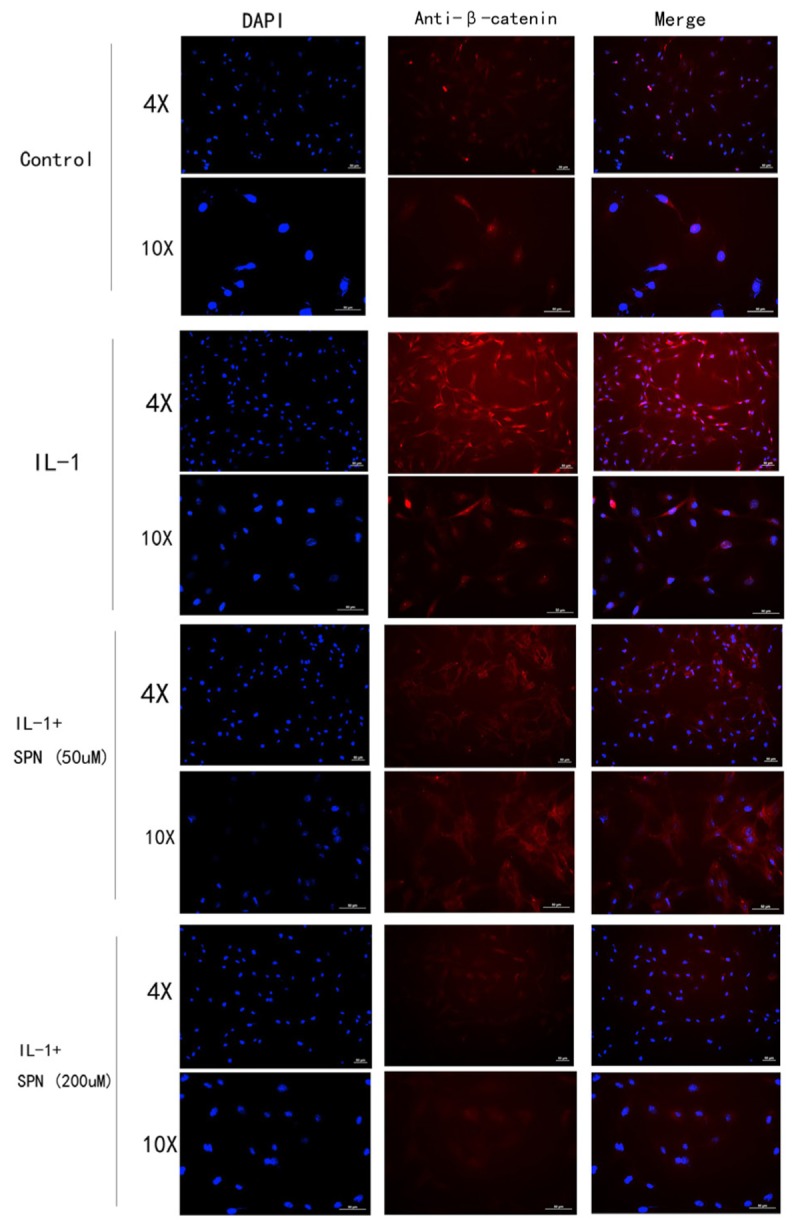
The location and concentration of β-catenin (red) was visualized using immunofluorescence microscopic analysis. Chondrocytes were treated with different concentrations of SPN for 6 h, with or without IL-1β (5 ng/ml) (*n* = 3 per group).

Subsequently, the location and concentration of NF-κB-p65 and β-catenin were visualized using immunofluorescence microscopic analysis. Chondrocytes were treated with SPN (200 μM) for 1 h, and then stimulated with IL-1β (5 ng/ml) for 30 min before NF-κB-p65 analysis, and for 6 h before β-catenin analysis. Inhibition of the two signaling pathways was observed (**Figures 6**, **7**). Therefore, we selected 200 μM as the concentration of SPN for all subsequent experiments.

### Effect of SPN on IL-1β-Induced Nuclear Translocation of NF-κB p65 and β-Catenin *in Vitro*

According to previous studies, the activation of NF-κB and wnt/β-catenin signaling involves the nuclear translocation of NF-κB p65 and β-catenin (Perkins, 2006; Clevers and Nusse, 2012). Here, to further investigate the effect of SPN on the IL-1β-induced activation of NF-κB and wnt/β-catenin signaling, we used Western blotting to observe the nuclear translocation of NF-κB p65 and β-catenin during the treatment. Chondrocytes were treated with two concentrations (0 or 200 μM) of SPN for 6 h, with or without IL-1β (5 ng/ml). According to **Figures 5A,B**, SPN treatment (200 μM) reduced the nuclear protein level of NF-κB p65 and β-catenin, whereas no significant difference was observed in the cytoplasmic protein level of the two proteins. Our results confirmed the nuclear translocation of NF-κB p65 and β-catenin in IL-1β-induced inflammation and showed that SPN can disrupt this process.

### Effect of SPN Treatment on Cartilage Degeneration in a Rat Model of OA

**FIGURE 8:**
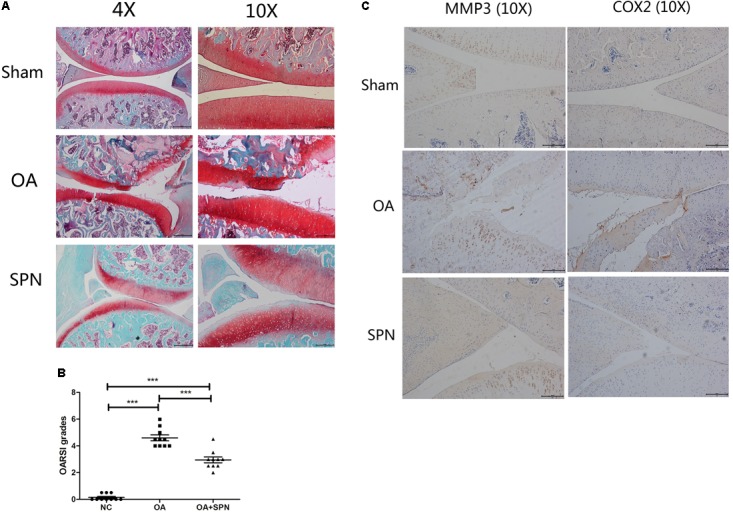
Effect of SPN treatment on cartilage degeneration in a rat model of OA. ACL and MM-transected rats were injected intra-articularly with SPN (200 μM) every 7 days. **(A)** Rats were sacrificed to isolate the knees for analysis after 8 weeks of treatment. 4% paraformaldehyde-fixed knees were decalcified and embedded in paraffin, then sectioned at 5 μm thickness. Sections of the interior joint were stained with safranin O-fast green (SO). **(B)** The OARSI grading system (0–6) was used to evaluate the sections. **(C)** Inflammatory factors (MMP3 and COX2) were detected by immunohistochemistry (*n* ≥ 10 per group). Significance was calculated by one-way ANOVA with *post hoc* Tukey’s multiple comparisons test. ^∗∗∗^*p* < 0.001.

As SPN treatment could protect the cartilage by down-regulation of IL-1β-induced expression of MMPs *in vitro*, we also investigated this effect *in vivo*. We developed an OA model in SD rats by surgical transaction of ACL and MM. Rats in the SPN group were injected intra-articularly with 200 μl SPN solution (200 μM, 0.14 mg/kg) every 7 days, starting at 1 week post-surgery, whereas rats in the OA group received equal volumes of vehicle. All rats were sacrificed after 8 weeks. OASRI based histological assessment was used to evaluate the OA grade. SO stained sections of knee joints in OA rats showed disrupted and discontinuous cartilage, for which the mean OARSI grade was 4.6. Meanwhile, the average OARSI grade of the SPN group was 2.95, as illustrated in **Figures 8A,B**, which was significantly lower than that of the OA group. Immunohistochemistry data (MMP3 and COX2, **Figure 8C**) also showed decreased levels of inflammatory factors in the SPN group compared with the OA group. Taken together, these results demonstrate that SPN treatment plays a cartilage-protective role in a rat model of OA.

### Effect of SPN Treatment on Degeneration of Trabecular Microarchitecture of Subchondral Bone in OA Rats

**FIGURE 9:**
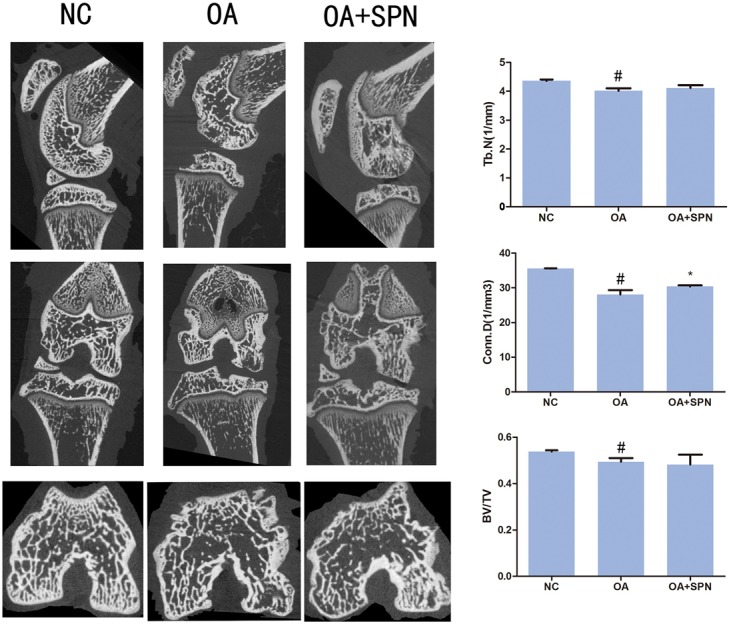
Effect of SPN treatment on degeneration of trabecular microarchitecture of subchondral bone in OA rats. The treatment was performed as described in **Figure 5**, and the knees were subjected to μCT for evaluation of subchondral bone. The CT Analyzer software was used to evaluate BV/TV, Tb.N., and Conn.D (*n* ≥ 10 per group). Significance was calculated by one-way ANOVA with *post hoc* Tukey’s multiple comparisons test. ^∗^*p* < 0.05 *versus* the OA group; ^#^*p* < 0.05 *versus* the NC group.

It is well-known that degenerative changes in the subchondral region are usually accompanied by degeneration in the articular cartilage (Li G. et al., 2013). Transection of ACL and MM changes the biomechanical environment in the joint and results in the development of OA. In the present study, μCT was used to investigate changes in the femoral subchondral bone. For this purpose, the trabecular bone volume fraction (BV/TV; %), trabecular number (Tb.N.; 1/mm), and connectivity density (Conn.D) were evaluated (**Figure 9**). In OA rats, the trabecular BV/TV, Tb.N, and Conn.D were all lower than those of the sham control (NC). These data confirm the presence of degenerative changes in the subchondral region in OA. Furthermore, we also investigated the effect of SPN treatment on these changes. The results showed that SPN treatment did not significantly improve the degenerative changes assessed by trabecular BV/TV and Tb.N, but the Conn.D of SPN-treated OA rats was significantly higher than that of control rats. These data suggest that SPN treatment might preserve the femoral subchondral bone microarchitecture in OA.

## Discussion

Nonsteroidal anti-inflammatory drugs (NSAIDs) have long been used in the treatment of OA in clinical practice. Although most NSAIDs can relieve symptoms such as pain and swelling, these drugs cannot prevent the degeneration of cartilages and trabecular microarchitecture of subchondral bone in OA. Moreover, long-term use of NSAIDs could result in a range of serious side effects. SPN is an extracted agent of the fruits of *Ligustrum lucidum*, which has only been known for the past few decades. To date, its pharmaceutical value was only studied in the neural system (Kawaguchi, 2009) and in the treatment of diabetic retinopathy (Cheleschi et al., 2017). To the best of our knowledge, we report for the first time that SPN could prevent OA-associated degeneration by reducing the activation of NF-κB and wnt/β-catenin signaling.

Cartilage degradation in OA is mediated by the MMP family (Troeberg and Nagase, 2012). High level of MMPs is regarded as one of the main expressions of OA (Tetlow et al., 2001; Tchetverikov et al., 2005; Li W. et al., 2013), combined with low levels of chondrocyte-specific proteins like sox9 and collagen II (Matyas et al., 2002; Zhang et al., 2015). Thus, regulating the expression of MMPs is a promising strategy for preventing OA. Firstly, we observed that SPN treatment at different concentrations (0, 10, 50, 100, 200 μM) does not affect the viability of rat chondrocytes, and does not alter gene expression under non-pathological conditions, which means that SPN does not significantly affect the normal cartilage. SPN decreased IL-1β-induced expression of matrix-degrading enzymes including MMP-3, MMP-9, IL-6, iNOS, and COX2. Furthermore, SPN treatment increased the protein level of sox9 and collagen II in IL-1β-induced inflammation. This suggests that SPN exerts its protective action by decreasing the expression of matrix-degrading enzymes, as well as by reducing chondrocyte-specific gene degradation induced by IL-1β in rat chondrocytes.

The NF-κB and wnt/β-catenin signaling pathways are two major pathways involved in OA development (Marcu et al., 2010; Ma et al., 2012). When the NF-κB pathway is activated by inflammatory mediators like IL-1β, NF-κB-p65 in the cytoplasm is translocated into nucleus and up-regulates multiple inflammation-related genes, such as MMPs, COX-2, and PGE2, thus NF-κB is a therapeutic target in OA (Roman-Blas and Jimenez, 2006). According to the results of Western blotting and immunofluorescence microscopy, the SPN treatment markedly reduced both the cellular level and the nuclear translocation of NF-κB-p65 caused by IL-1β stimulation. Moreover, SPN treatment significantly inhibited IL-1β-induced phosphorylation of NF-κB-p65. When the wnt/β-catenin pathway is inactive, β-catenin is targeted for proteasomal degradation through the β-TrCP/Skp pathway (MacDonald et al., 2009). Thus, in this case, the β-catenin level remains low because of continuous degradation. When the wnt/β-catenin pathway is activated, β-catenin becomes stable as proteasomal degradation is reduced, and stabilized β-catenin is then translocated into the nucleus up-regulating various inflammation-related genes like those encoding MMPs (MacDonald et al., 2009). Phosphorylation primes β-catenin for subsequent phosphorylation by GSK-3β at Ser33, Ser37, and Thr41, which destabilizes β-catenin (Amit et al., 2002; Liu et al., 2002). Consequently, in the present study, active (non-phospho) β-catenin was also assessed. As illustrated in **Figures 4**–**7**, SPN not only reduced IL-1β-induced β-catenin in the whole cell, but also inhibited the nuclear translocation of β-catenin. Taken together, our results indicate that SPN treatment inhibits IL-1β-induced inflammation by regulating the NF-κB and wnt/β-catenin pathways *in vitro*, but the exact target of SPN is still unknown. Several studies already indicated the crosstalk between NF-κB and wnt/β-catenin pathways (Ma and Hottiger, 2016), activation of β-catenin pathway could enhance βTrCP-mediated IκB degradation, meanwhile IKKα could also inhibit degradation β-catenin. The target of SPN could be either or both of these two pathways; we will study it deeper in following studies.

We further examined the *in vivo* effect of SPN in a rat model of OA. At present, OA progression is associated with changes in the microarchitecture of the subchondral bone combined with cartilage degeneration (Moodie et al., 2011). In the animal experiment, we evaluated degeneration in both the articular cartilage (**Figure 8**) and subchondral bone (**Figure 9**). In the OA group, erosion in the cartilage was observed and loss of a large mass of cartilage matrix was usual. In addition, deformation was also detected in some knees. In the SPN group, the structural integrity of the cartilage was maintained in most knees. OARSI grading results showed a significant protection of the articular cartilage by SPN. A previous study indicates that changes in the biomechanical environment, like ACL-transaction-caused instability of joint, could influence the subchondral bone, resulting in the concentration and increased activity of osteoclasts that can promote subchondral bone resorption (Zhen et al., 2013). In the present study, resorption of subchondral bone in OA rats was investigated *via* μCT analysis. The improvement in trabecular microarchitecture of femoral subchondral bone in the presence of SPN was also indicated by the measured connectivity density (Conn.D, **Figure 9**). Thus, our results suggest that SPN has a therapeutic effect in OA *in vivo*.

## Conclusion

In this study, we demonstrate, for the first time, that SPN decreases the expression of cartilage matrix-degrading enzymes and the activation of NF-κB and wnt/β-catenin signaling, while increasing chondrocyte-specific gene expression in IL-1β-induced inflammation in chondrocytes. Additionally, animal experiments showed that SPN plays a therapeutic role in OA by decreasing the degeneration of articular cartilage and subchondral bone.

## Materials and Methods

### Animals

Sprague-Dawley (SD) rats (200–250 g; 6 weeks old, Animal Center of Zhejiang University) were used in this study. Water and food were provided routinely in the facility. The study was conducted in accordance with the Declaration of Helsinki, and the protocol was approved by the Ethics Committee of The Second Affiliated Hospital, Zhejiang University School of Medicine, Hangzhou, China (2015-107).

### Materials

Specnuezhenide was obtained from Shanghai Pureone Biotechnology. Recombinant rat IL-1β was purchased from R&D Systems, Abingdon, United Kingdom. Dulbecco’s Modified Eagle’s Medium (DMEM), penicillin and streptomycin, FBS, and 0.25% trypsin were obtained from Gibco RRL, Grand Island, NY, United States. Collagenase II was purchased from Sigma-Aldrich, St. Louis, MO, United States.

### CCK-8 Assay

Cell counting kit-8 was used to evaluate the cytotoxicity of SPN according to the manufacturer’s instruction. Rat chondrocytes were seed in 96-well plates, at a density of 5000/well. After incubation with different concentrations of SPN (0, 10, 50, 100, 200 μM) for 24 h and 48 h, cells were incubated with 10 μl CCK-8 for 4 h. The optical density (OD) was measured at a wavelength of 450 nm with a reader.

### Cell Culture and Treatment

Rat chondrocytes were prepared as previously described (Li W. et al., 2013; Zhou et al., 2013). Briefly, 6-week-old SD rats were sacrificed, and cartilages from the hip joints were digested with 0.25% pancreatic enzymes for 30 min to remove other tissues and cells. Then, the cartilage fragments were digested with 0.2% collagenase II to isolate the cells. The chondrocytes were grown in DMEM with 10% FBS, 100 U/ml penicillin, and 100 mg/ml streptomycin, at 37°C, with 5% CO_2_. Cells were then seeded in six-well plates for analysis. Subconfluent cells were pretreated with SPN at different concentrations for 1 h, then incubated with or without IL-1β (5 ng/ml) for 24 h or 6 h, according to the study design.

### RNA Extraction and Real-Time PCR

We used TRIzol reagent (Invitrogen, Carlsbad, CA, United States) to extract RNA from cells according to the manufacturer’s protocol. Total RNA was used to synthesize cDNA by reverse transcription (cDNA synthesis kit, Takara). Power SYBR Green PCR Master Mix (Applied Biosystems) was used for real-time PCR. The expression of MMP3, MMP9, IL-6, iNOS, COX2, collagen II, and sox9 was analyzed using the primer sequences listed in **Table 1**. GAPDH was used as endogenous control. For real-time PCR, 10 μL reaction mixture contained SYBR Green and each primer. PCR was set using the StepOnePlus system (Applied Biosystems). The program included one cycle of denaturation at 95°C for 1 min and 40 cycles of denaturation at 95°C for 15 s, with subsequent primer annealing, and extension at 63°C for 25 s, followed by melt curve analysis. Data were analyzed for fold difference using the 2^-ΔΔC_T_^ method.

**Table 1:** Primers used for real-time PCR.

Gene^∗^	Forward	Reverse
Rat MMP3	CAGGCATTGGCACAAAGGTG	GTGGGTCACTTTCCCTGCAT
Rat MMP9	GCAAACCCTGCGTATTTCCAT	GATAACCATCCGAGCGACCTTT
Rat collagen II	GAGTGGAAGAGCGGAGACTACTG	GTCTCCATGTTGCAGAAGACTTTCA
Rat sox9	CCAGCAAGAACAAGCCACAC	CTTGCCCAGAGTCTTGCTGA
Rat COX2	GAGAGATGTATCCTCCCACAGTCA	GACCAGGCACCAGACCAAAG
Rat iNOS	CCTTACGAGGCGAAGAAGGACAG	CAGTTTGAGAGAGGAGGCTCCG
Rat IL-6	AGCGATGATGCACTGTCAGA	GGAACTCCAGAAGACCAGAGC
Rat GAPDH	GAAGGTCGGTGTGAACGGATTTG	CATGTAGACCATGTAGTTGAGGTCA

^∗^*Rat means species is rat*.

### Protein Extraction and Western Blot Analysis

Radioimmunoprecipitation assay (RIPA) containing protease and phosphatase inhibitors was used to prepare whole cell extracts after treatment. Nuclear and Cytoplasmic Extraction Reagents (Bosterbio, Wu Han, China) were used to prepare respective extracts. Equal amounts of cell extract were separated by 10% SDS-PAGE, and electro-transferred to polyvinylidene difluoride membranes. After blocking with 5% Bull Serum Albumin (BSA, Sigma-Aldrich, St. Louis, MO, United States) for 2 h, the membranes were blotted with primary Ab at 4°C overnight, then incubated for 1 h with secondary antibody. In the present study, Ab against MMP-3 (rabbit mAb, Abcam, ab52915), MMP-9 (rabbit mAb, Abcam, ab76003), IL-6 (10E5, mouse mAb, Santa Cruz, sc-57315), iNOS (rabbit mAb, Abcam, ab3523), COX2 (D5H5, rabbit mAb, Cell Signaling, #12282), collagen 2 (rabbit mAb, Abcam, ab188570), sox9 (rabbit mAb, Abcam, ab185966), β-catenin (D10A8, rabbit mAb, Cell Signaling, #8480p), non-phospho (active) β-catenin (Ser45) (D2U8Y, rabbit mAb, Cell Signaling, #19807S), NF-κB p65 (C22B4, rabbit mAb, Cell Signaling, #4764S), phosphor-NF-κB p65 (Ser536) (rabbit Ab, Cell Signaling, #3031), β-actin (mouse mAb, Abcam, ab8226), GAPDH (rabbit mAb, Cell Signaling, #5174), and TBP (rabbit Ab, Bosterbio, BA3586-2) were used. GAPDH and β-actin worked as endogenous controls for whole cell and cytoplasmic protein assessment. TBP worked as endogenous control for nuclear protein analysis.

### Immunofluorescence Microscopy

Co-expression of key proteins of wnt/β-catenin and NF-κB pathways was carried out using fluorochrome-conjugated Ab. Chondrocytes cultured on glass coverslips were fixed in 4% paraformaldehyde for 10 min, and permeabilized for 5 min with 0.1% v/v Triton X-100. Cells were incubated with primary antibody at 4°C overnight, washed, and then incubated with fluorochrome-conjugated secondary antibody for 2 h in the dark. Coverslips were mounted onto glass slides using DAPI-containing mounting medium.

### Luciferase Reporter Gene Analysis

Chondrocytes were first stably transfected with a luciferase reporter construct (NF-κb-Luc or TCF-LEF RE, Promega, Madison, WI, United States). Then, transfected cells were pretreated with various concentrations of SPN (0, 50, 100, 200 μM) for 1 h and then stimulated with IL-1β (5 ng/ml) for 6 h for NF-κb-Luc activity analysis, or 12 h for TCF-LEF RE activity analysis. Subsequently, the luciferase assay system (Promega) was used to measure luciferase activity.

### Induction of OA in Rats

The rat model of OA was developed by surgical excision of ACL and MM on the knee joints. In brief, SD rats were anesthetized by pentobarbital (40 mg/kg). A medial para-patellar approach was used here, for which the patella was retracted laterally to expose the MM and ACL. The ACL was transected and the MM was removed. The operational wound was closed in layers by sutures.

Overall, 45 rats were used in the present study, randomly divided into three groups. Rats in the control group received sham surgery to act as sham control (NC). For the treatment group (SPN group), 200 μl SPN solution (200 μM, 0.14 mg/kg) were injected intra-articularly in OA rats every 7 days starting at 1 week post-surgery, whereas rats in the OA group received an equal volume of vehicle. All rats were sacrificed after 8 weeks of treatment, and the knees were preserved in 4% paraformaldehyde solution.

### μCT Analysis

μCT analysis was conducted using a μCT system (vivaCT80, Scanco Medical, Zurich, Switzerland) to evaluate the structure of subchondral bone with the following settings: X-ray 50 kV, 200 μA; integration time, 300 ms; slice thickness, 0.02 mm. Reconstruction of the sections was done by MicView software. The region between the articulating surface of condyles and the growth plate was assessed. Various trabecular bone indices such as trabecular bone volume fraction (BV/TV; %), trabecular number (Tb.N.; 1/mm), and connectivity density (Conn.D) were evaluated using the CT Analyzer software.

### Histological Analysis

4% paraformaldehyde solution fixed knees were decalcified and embedded in paraffin, followed by sectioning at 5 μm thickness. The sections of interior joint were stained with SO. The OARSI grading system (0–6) was used to evaluate the sections (Pritzker et al., 2006).

### Immunohistochemical Analysis

Sections from each group were immunohistochemically stained for assessment of OA. Ab against MMP-3 (Abcam, ab52915) and COX2 (Cell Signaling, #12282) were used in this analysis.

### Statistical Analysis

The results are presented as mean ± standard deviation of three experiments. Statistical differences were determined with SPSS 12.0. One-way ANOVA with subsequent *post hoc* Tukey’s test was used for multiple comparisons. *P* < 0.05 was considered indicative of statistical significance.

## Author Contributions

All authors listed have made substantial contributions to the study. LW, HW, CM, XZ, and KX took part in the designing of the experiments, contributed reagents, materials, and analysis tools. CM, XZ, KX, LW, YY, and JR run the experiments. CM and JR wrote the manuscript. LW and AL also participated in the analyzing of the data. All authors were involved in the study and preparation of the manuscript. All authors read and approved the final manuscript.

## Conflict of Interest Statement

The authors declare that the research was conducted in the absence of any commercial or financial relationships that could be construed as a potential conflict of interest.

## Abbreviations

Ab: antibodies
ACL: anterior cruciate ligament
CCK-8: cell counting kit-8
COX2: cyclooxygenase-2
FBS: fetal bovine serum
GSK-3β: glycogen synthase kinase-3 beta
IL-1β: interleukin-1β
IL-6: interleukin-6
iNOS: inducible nitric oxide synthase
mAb: monoclonal antibodies
MM: medial meniscus
MMP: matrix metalloproteinase
NF-κB: nuclear factor-κB
OA: osteoarthritis
OARSI: The Osteoarthritis Research Society International
SO: safranin O-fast green
SPN: specnuezhenide
TBP: TATA-box binding protein
TNF-α: tumor necrosis factor alpha.
