# Supplementary material for: Specnuezhenide Decreases Interleukin-1β-Induced Inflammation in Rat Chondrocytes and Reduces Joint Destruction in Osteoarthritic Rats
Source: Front Pharmacol. 2018 Jun 28;9:700. doi: 10.3389/fphar.2018.00700 (PMC6052343; doi:10.3389/fphar.2018.00700)
Supplement: Supplementary file 1 [file Data_Sheet_1.DOCX]

Methods in supplementary part

CCK-8 assay

RAW264.7 cells were used here. To evaluate the cytotoxicity of SPN, cells were seeded in 96-well plates, at a density of 5000/well. After incubation with different concentrations of SPN (0, 10, 50, 100, 200 μM) for 24 h, cells were incubated with 10 μl CCK-8 for 4 h. The optical density (OD) was measured at a wavelength of 450 nm with a reader.

Cell culture and treatment

RAW264.7 cells were incubated in cell culture (α-MEM containing 10% FBS, 100 U/mL penicillin, and 100 mg/mL streptomycin). When When the cells reached 90% confluence, cells were washed with PBS and trypsinized at 37˚C with 5% CO2 for 2 min for harvest. The harvested cells were seeded in plates for analysis Subconfluent cells were pretreated with SPN at different concentrations for 1 h, then incubated with or without LPS (10 ng/ml, Sigma-Aldrich) for 24 h before RT-PCR and Western blot.

RT-PCR

The expression of INOS and COX2 was analyzed using primers sequences as followed:

GAPDH: forward, 5’-AGGGCGCCTTCTCTTGTGAC-3’ and reverse, 5’-TGGGTAGAATCATACTGGAACATGTAG-3’;

iNOS: forward, 5’- AGCGGCCCATGACTCTCA-3’ and reverse, 5’-CTGCACCCAAACACCAAGGT-3’;

COX-2: forward, 5’- CCTTGAAGACGGACTTGCTCAC-3’ and reverse, 5’-TCTCTCTGCTCTGGTCAATGGA-3’;

GAPDH was used as endogenous control. Methods of RNA extraction and RT-PCR are already described previously

Figure legend

Figure S1. Effect of SPN treatment on LPS-induced inflammation in RAW264.7 cells. (a) cell viability was assessed by CCK-8 assay (n=5, per group). RAW264.7 cells were pretreated with SPN at different concentrations (0, 50, 100, 200 μM) for 1 h, then incubated with or without LPS (10 ng/ml) for 24 h. (b) The expression of INOS and COX2 was analyzed by RT-PCR (n=3, per group). (c) Protein level of INOS and COX2 was analyzed by Western blot (n=3, per goup). Significance was calculated by one-way ANOVA with post hoc Tukey’s multiple comparisons test. *p < 0.05, **p < 0.01, ***p < 0.001 *versus* the LPS+SPN (0 μM) group.
