# Supplementary figures and images for: Specnuezhenide Decreases Interleukin-1β-Induced Inflammation in Rat Chondrocytes and Reduces Joint Destruction in Osteoarthritic Rats
Source: Front Pharmacol. 2018 Jun 28;9:700. doi: 10.3389/fphar.2018.00700 (PMC6052343; doi:10.3389/fphar.2018.00700)

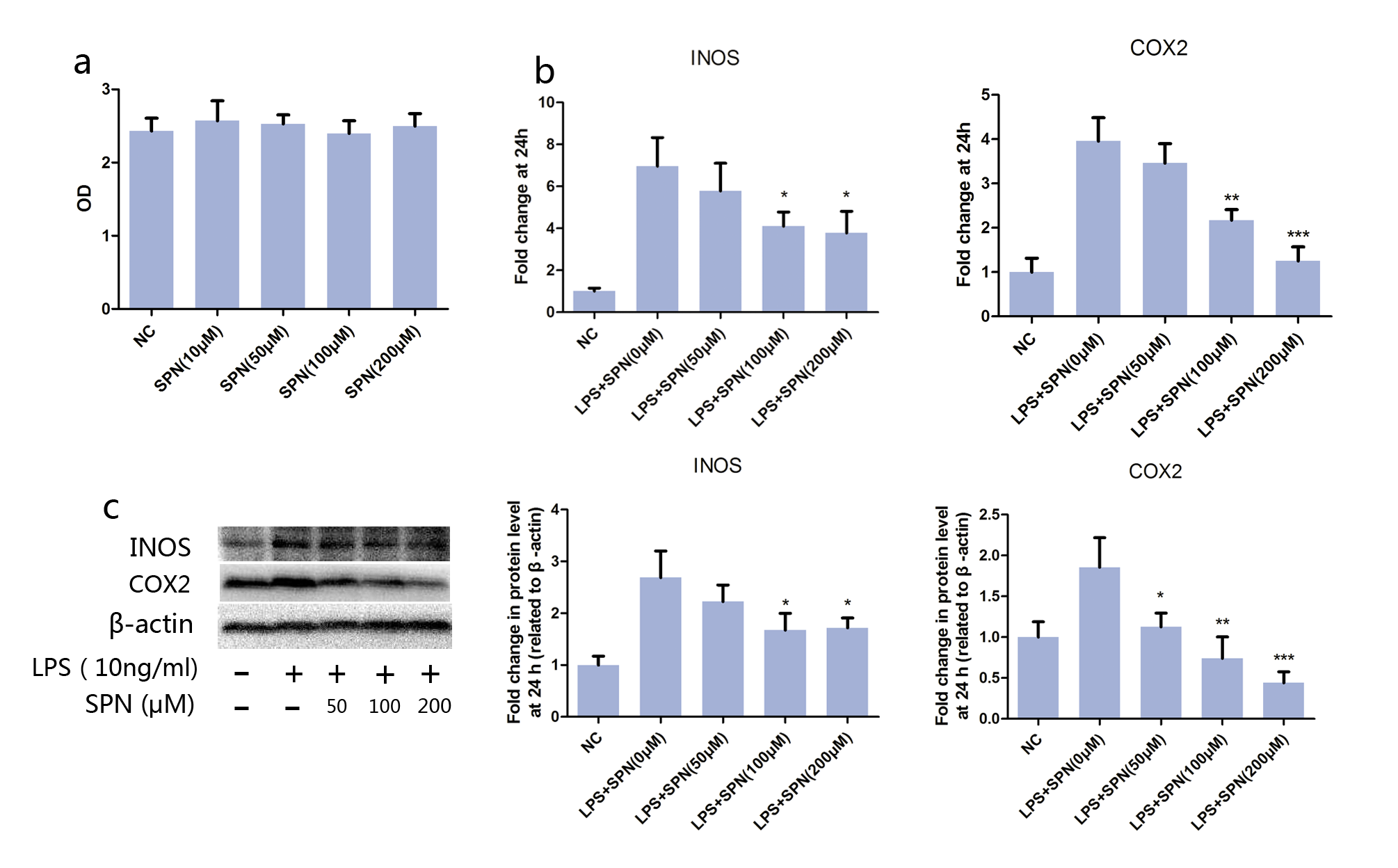

Supplement: Supplementary file 2 [file Image_1.TIF]
